# Clinico-Pathological Importance of miR-146a in Lung Cancer

**DOI:** 10.3390/diagnostics11020274

**Published:** 2021-02-10

**Authors:** Javaid Ahmad Wani, Sabhiya Majid, Andleeb Khan, Azher Arafah, Ajaz Ahmad, Basit Latief Jan, Naveed Nazir Shah, Mohsin Kazi, Muneeb U. Rehman

**Affiliations:** 1 Department of Biochemistry, Government Medical College (GMC-Srinagar), Karan Nagar, Srinagar 190010, Jammu and Kashmir, India; wanijavaidstd@gmail.com (J.A.W.); zululubaba@gmail.com (S.M.); 2 Department of Pharmacology and Toxicology, College of Pharmacy, Jazan University, Jazan 45142, Saudi Arabia; drandleebkhan@gmail.com; 3 Department of Clinical Pharmacy, College of Pharmacy, King Saud University, Riyadh 11451, Saudi Arabia; aazher@ksu.edu.sa (A.A.); bjan@ksu.edu.sa (B.L.J.); 4 Department of Pulmonary Medicine, Government Medical College (GMC-Srinagar), Karan Nagar, Srinagar 190010, Jammu and Kashmir, India; naveednazirshah@yahoo.com; 5 Department of Pharmaceutics, College of Pharmacy, King Saud University, Riyadh 11451, Saudi Arabia; mkazi@ksu.edu.sa

**Keywords:** miR-146a, lung cancer, biomarker, clinical pathology

## Abstract

Lung cancer is a well-known malignant tumor of the respiratory tract, which has caused a significant level of damage to human health in the 21st century. Micro-RNAs (miRNAs) are tiny, non-coding RNA stem-loop structures with a length of roughly 20–25 nucleotides that function as powerful modulators of mRNA and protein products of a gene. miRNAs may modulate many biological processes involving growth, differentiation, proliferation, and cell death and play a key role in the pathogenesis of various types of malignancies. Several accumulating pieces of evidence have proven that miRNA, especially miR-146a, are crucial modulators of innate immune response sequences. A novel and exciting cancer research field has involved miRNA for the detection and suppression of cancer. However, the actual mechanism which is adopted by these miRNA is still unclear. miRNAs have been used as a cancer-associated biomarker in several studies, suggesting their altered expression in various cancers compared to the normal cells. The amount of expression of miRNA can also be used to determine the stage of the disease, aiding in early detection. In breast, pancreatic, and hepatocellular carcinoma, and gastric cancer, cancer cell proliferation and metastasis has been suppressed by miR-146a. Changes in miR-146a expression levels have biomarker importance and possess a high potential as a therapeutic target in lung cancer. It retards epithelial-mesenchymal transition and promotes the therapeutic action of anticancer agents in lung cancer. Studies have also suggested that miR-146a affects gene expression through different signaling pathways viz. TNF-α, NF-κB and MEK-1/2, and JNK-1/2. Further research is required for understanding the molecular mechanisms of miR-146a in lung cancer. The potential role of miR-146a as a diagnostic marker of lung cancer must also be analyzed. This review summarizes the tumor-suppressing, anti-inflammatory, and antichemoresistive nature of miR-146a in lung cancer.

## 1. Introduction

Lung cancer is one of the most common malignancies worldwide (11.6%) and accounts for nearly 18.5% of cancer-related mortality. About 2,093,876 new cases of lung cancer are diagnosed per year, with 1,761,007 deaths [1]. Small cell lung cancer (SCLC) is characterized as a neuroendocrine carcinoma because the cancer cells have features of nerve cells and endocrine (hormone-secreting) cells and is less prevalent (16.8%) but more lethal and heterogeneous than non-small cell lung cancer (NSCLC) among the population (80.4%) [2]. Lung carcinogenesis is a multifactorial and multistep process that causes sequential accumulation of molecular and genetic defects, especially because of tobacco use. It initiates with the loss of 9p and 3p chromosomes and ends with cyclin D1 and E overexpression [3,4,5]. Takamizawa et al. first observed non-coding RNA dysregulation in lung cancer when he noticed a significantly reduced level of let-7 associated with the post-operative survival of lung cancer patients [6]. Lin-4 became the first miRNA discovered when R. C. Lee was studying postembryonic developmental mechanisms in *C. elegans* [7]. These are a group of small non-coding RNAs with an excellent function in post-transcriptional regulation of gene expression. The two ways by which miRNA monitors gene expression are by arresting mRNA translation or by mRNA degradation [7]. Since malignancy accounts for highly heterogenous diseases, analysis of miRNA expression can discriminate among subtypes of cancer [8,9,10,11] and has the potential to detect the unknown origin site of primary cancer [12]. miRNAs can serve as good candidates to predict the clinical outcome or clinical progression of cancer, including its survival rate, severity [13], and development of chemoresistance [14,15,16].

Dr. David Baltimore found that the gene for miR-146a was located on chromosome 5 [17,18]. miR-146a was found to facilitate programmed cell death and discourage cell proliferation and cell migration in NSCLC cell lines that were the key hallmarks of cancer [19]. miR-146a also showed downexpression, which was proven in several NSCLC cell lines [20] and significantly associated with advanced-stage lung cancer that lowered progression-free survival [19]. The present review will focus on the potential role of miR-146a as a tumor-suppressive, anti-inflammatory, diagnostic, and prognostic tool in lung cancer.

## 2. miR-146a Expression and Regulation

### 2.1. Biogenesis

**Figure 1:**
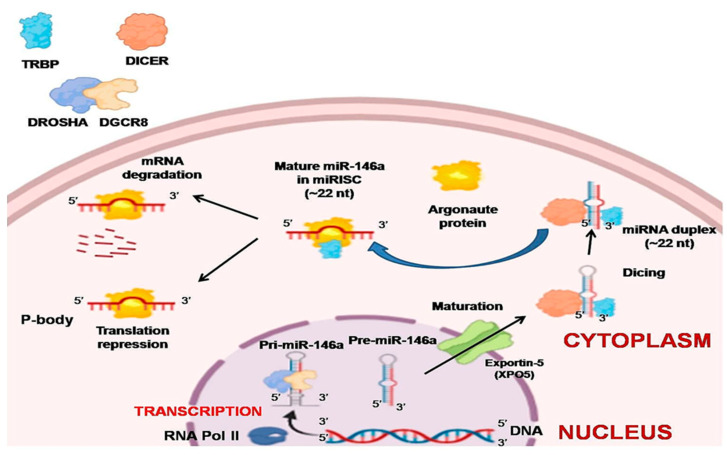
Biogenesis of miR-146a. Pri-miR-146a is formed by RNA polymerase II, which is further processed by capping, splicing, and polyadenylation. The long pre-miR-146a is cleaved by the microprocessor to release a hairpin-shaped pre-miR-146a. This leaves the nucleus through exportin 5 (XPO5) into the cytoplasm. In the cytoplasm, DICER with transactivation-responsive RNA-binding protein (TRBP) further acts on the pre-miR-146a to generate miRNA duplex. Argonaute protein selectively binds to one of the strands of miRNA, and a miRNA-induced silencing complex (miRISC) assembly is formed. The strand to which argonaute binds serves as a guide strand and leads the complex to complementary target mRNAs with GW182 protein family members for post-transcriptional gene silencing forming processing bodies (P-bodies). The other fate of miRNA is degradation if it is not needed.

The regulatory role of miRNAs was not well known until 2001, after which thousands of miRNAs in diverse kinds of species were discovered [6,21]. RNA polymerase II (Pol II) is the main enzyme that catalyzes most of the miRNA gene transcription from the dedicated miRNA gene loci in the nucleus, and around 30% are generated from protein-coding gene introns. The resultant primary miRNAs (pri-miRNAs) are further processed by capping, splicing, and polyadenylation [7]. A single miRNA or a group of miRNAs are produced by these pri-miRNAs from a common primary transcript. The long pre-miRNAs are cleaved by microprocessors, which are a complex of dsRNase III enzyme (DROSHA) and a cofactor called double-stranded RNA (dsRNA) binding protein vital region 8 (DGCR8) [22,23]. The two dsRNA strands of pri-mi-RNAs are cleaved toward the base of secondary stem-loop structures by the two RNA-III domains found in DROSHA, which release hairpin-shaped precursor miRNAs (pre-miRNAs; ~60–70 nucleotide) [23,24,25]. The single-stranded RNA (ssRNA) stem junction and the distance from the terminal loop region are then recognized by the microprocessor. In particular, the microprocessor slices the dsRNA at ~10–12 bp from the juncture with flanking ssRNA, thereby creating hairpin-shaped pre-miRNAs with an overhang of either two nucleotides (group I miRNAs) or one nucleotide (group II miRNAs) at the 3′ end [26,27,28,29]. Since the essential elements, DROSHA and DGCR8, are necessary for the biogenesis of virtually all cell’s miRNAs, in vitro microprocessor activity contained in recombinant DROSHA and DGCR8 proteins can be reconstituted [30]. Many auxiliary elements are believed to play a role in the production of cells’ pri-miRNA. Exportin 5 (XPO5) then transports the pre-miRNAs to the cytoplasm from the nucleus. This is further processed by DICER1, an RNase III enzyme measuring the 5′ and 3′ pre-miRNA ends. DICER1 binds to the end of the pre-miRNA and positions its two catalytic RNase III domains such that the mature ~22-nucleotide miRNA duplex with 2-nucleotide 3′ overhangs is formed by asymmetrical cleavage of the dsRNA stem close to the terminal loop sequence. Transactivation-responsive RNA-binding protein (TRBP; also known as TARBP2) that binds to dsRNA is then associated with DICER1 [31,32]. While DICER1 does not involve pre-miRNA processing, TRBP increases the accuracy of DICER1-mediated cleavage of pre-miRNAs in a structure-dependent manner and modifies the selection of miRNA guide strands by leading to iso-miRNA formations that are one nucleotide longer than the normal miRNAs [31]. The other function of TRBP is to bridge DICER1 and argonaute proteins (AGO1, AGO2, AGO3, or AGO4) physically to participate in the miRNA-induced silencing complex (miRISC) assembly [32]. Argonaute protein binds to one strand of the mature miRNA, which is the guide strand, and leads the complex to target complementary mRNAs with GW182 protein family members for post-transcriptional gene silencing. This happens in processing bodies (P-bodies) where the cytoplasmic foci are induced by mRNA silencing and decay but are not usually necessary for the silencing of miRNA-mediated genes [33,34] (Figure 1).

### 2.2. Transcriptional and Post-Transcriptional Regulation

Throughout vertebrate species, miR-146a (mature) is greatly conserved [35]. As discussed above, the pri-miRNA-146a is produced as an independent unit that is regulated by a unique regulatory sequence. As shown by the experimentation of Taganovet et al. (2006), this regulatory sequence is located 16 kb upstream from the miR-146a gene. This regulatory sequence behaves as an interacting sequence for specific transcription activators or repressor factors as one site for C/EBPβ, IRF3/7, and two for NF-kB. Any kind of inflammatory signaling pathway that is mediated via the NF-kB factor strongly upregulates miR-146a expression such as lipopolysaccharide (endotoxin) from Gram-negative bacteria, TNF-α, and IL-1β signaling pathways [18]. In melanoma cells, apart from NF-kB, researchers also found that the c-MYC site increases miR-146a expression [36,37].

**Figure 2:**
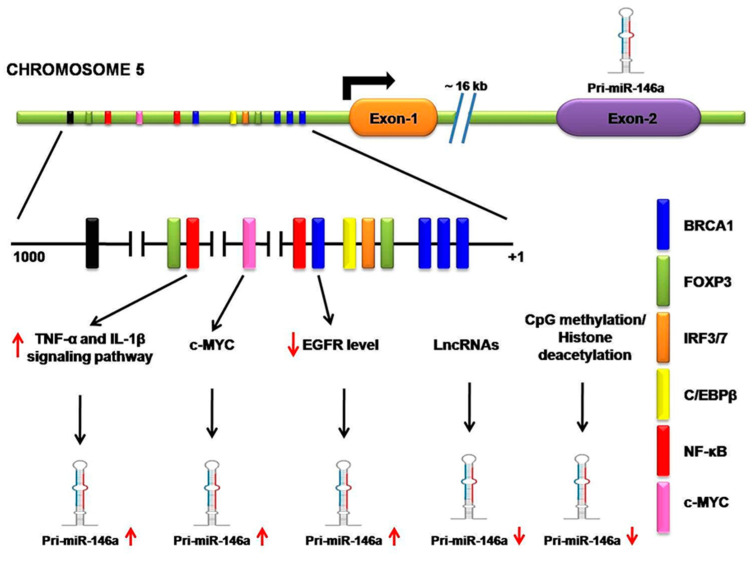
Transcriptional and posttranscriptional regulation of miR-146a. A regulatory sequence is located 16 kb upstream from the miR-146a gene. It contains a site for several transcription activator or repressor factors, such as breast cancer 1(BRAC1), forkhead box protein P3 (FOXP3), interferon regulatory transcription factor (IFR3/7), CCAAT enhancer-binding proteins β (C/EBPβ), nuclear factor kappa-B (NF-κB), and c-MYC. NF-κB regulatory inflammatory signaling pathways strongly upregulate miR-146a. c-MYC also increases miR-146a expression. BRCA1 directly binds the miR-146a promoter, increasing its expression, which in turn represses EGFR levels. Long non-coding RNA (lncRNA) directly interacts with miR-146a and significantly interferes with its gene-silencing function. CpG methylation on the miR-146a regulatory sequence causes its downregulation.

Molecular mechanisms, such as CpG methylation in the promoter or histone deacetylations, lead to downregulation of miR-146a in cancers [38]. For instance, CpG methylation on the miR-146a regulatory sequence causes its downregulation in NSCLC cell lines [20], which is also observed in hepatocellular carcinoma (HCC) [39] and prostate cancer cells [40]. H3 histone deacetylation at NF-kB sites leads to downregulation of miR-146a in macrophages and experiences significant improvement in miR-146a expression when treated with HDAC inhibitor. It is interesting to note that long non-coding RNA (lncRNA) directly interacts with miR-146a and significantly interferes with its gene-silencing function since lncRNA possesses multiple miRNA binding sites that trap miRNA and prevent its biological function [41]. For instance, lncRNA NIFK-AS1 in tumor-associated macrophages (TAMs) traps miR-146a and promotes Notch1 signaling in endometrial malignancies [42]. According to a similar study, in which lncRNAs like SNHG16, whose upregulation causes miR-146a downregulation, shows lncRNA also facilitates the progression of NSCLC [43]. Kumaraswamy et al. found the restoration of BRCA1 expression in breast cancer significantly decreases EGFR levels as well as restores miR-146a expression. It is confirmed that BRCA1 directly binds the miR-146a promoter and increases its expression, which in turn represses EGFR levels [44]. By studying malignant breast cells (MCF7) and normal epithelial breast cells (MCF10A), Lie et al. observed forkhead box protein P3 (FOXP3) binding sites in the miR-146a promoter that regulates its expression at the transcriptional level [45]. One study based upon chromatin immunoprecipitation (ChIP) suggests that the signal transducer and activator of transcription 3 (STAT3) directly binds to the promoter region of miR-146a, but no relevant binding site positions are described by the authors [46]. In summary, the expression of miR-146a is regulated by various regulatory proteins, especially transcription factors, as well as by epigenetic modifiers and lncRNA in various cells. These experiments have demonstrated that, in different types of cells, various regulatory proteins and transcription factors are responsible for miR-146a regulation. However, modifications and alterations of these mechanisms in cancer cells contribute to the misexpression of miR-146a. When Larner-Svensson et al. used pharmacological inhibitors on human airway smooth muscle cells, he observed that the expression of IL-1β-induced miR-146a was controlled at the transcriptional level by activating IKK2 (i.e., by NF-κB activity) but the pathway that produced mature miR-146a from the primary transcript was regulated by the mechanism-dependent modulation of MEK-1/2 and JNK-1/2 [47] (Figure 2).

## 3. Role of miR-146a in Lung Cancer

As observed by Bertoli and coworkers, different groups of miRNAs play an important role in different hallmarks of breast cancer [48]. It has been suggested that miR-146a may play an important role in several hallmarks of lung cancer, such as evading growth barricades, cell proliferation, resisting cell death, promoting angiogenesis, inflammation, tumor immune tolerance, energy metabolism imbalance (Warburg effect), metastasis, and genome fragility, that were first explained by Hanahan and Weinberg [49]. Since miRNA dysregulation has been found in almost every type of tumor, their involvement in cancer hallmarks is likely. Apart from lung cancer hallmarks, miR-146a involvement in cancer stem cells (CSCs) has also been observed. There is a similarity between EMT and CSC formation since both have several common signaling mediators like Notch, Wnt, and Hedgehog proteins. Many studies have found stem cell-like properties, thus sharing key signaling pathways and drug resistance phenotypes with CSCs [50,51].

### 3.1. miR-146a as an Antiproliferative and Proapoptotic Agent

Most of the research associated with miR-146a indicates its tumor-suppressive role in malignancies. In NSCLC cell lines and human lung tissue samples, the expression of this miRNA is strongly downregulated and it also displays antiproliferative and antiapoptotic properties in lung cancer cell lines [52,53]. The gene for EGFR found to be commonly mutated in lung adenocarcinoma patients, especially in nonsmoking women with Asian ethnicity (50%) [54,55], is directly targeted by miR-146a [19]. Qi et al. (2019) recently found that miR-146a-5p directly targets EGFR mRNA while studying the effect of cryptotanshinone derived from *Salvia miltiorrhiza* on NSCLC. It was also observed that cryptotanshinone prevents cell cycle progression by upregulating miR-146a/b levels [56]. The tumor collagenase stimulatory factor, also known as the extracellular matrix metalloproteinase inducer (EMMPRIN), is expressed on the outer surface of human tumor cells. This glycoprotein interacts with the fibroblasts and triggers many matrix metalloproteinase expressions within the fibroblasts [57]. Huang WT et al. proved through various study methods like TargetScan, luciferase enzyme assay, cancer genome atlas, and immunohistochemistry that miR-146a directly targeted the tumor collagenase stimulatory factor (TCSF) to modulate the mechanisms of cell viability and apoptosis in NSCLC [58]. Macrophage migration inhibitory factor (MIF) was considered an important cytokine for the regulation of innate immunity. Thorsten Hagemann et al. knocked down either EMMPRIN or MIF and found decreased invasiveness and matrix metalloproteinase activity in the supernatant of tumor cell culture [59]. MIF was also found to be the promoter of the Warburg effect in NSCLC by activating NF-kB/HIF-1α inflammatory signaling [60,61]. The macrophage migratory inhibition factor (MIF) gene upregulated in NSCLC is the reverse target of miR-146a, as proven by luciferase assay mimic studies in A549 cells, and promotes apoptosis and discourages proliferation [62]. Another similar study found cell cycle progression was also slowed down by miR-146a via specifically downregulating the expression of CCND1/2 (genes of cyclins D1/2), both at post-transcriptional and protein stages that arrested the G0/G1 phase of cell cycle progression [63] and targeted cyclin J that promoted NSCLC chemosensitivity to cisplatin [64]. It was found that miR-146a overexpression maintained epithelial phenotypes and discouraged epithelial to mesenchymal transition in lung cancer cell lines by suppressing insulin receptor substrate-2 (IRS2) transcription and translation [65]. It has also been found that IRS2 enhances the Wnt/β-catenin pathway, thus increasing N-cadherin but decreasing E-cadherin [66]. This was further confirmed by a study of a xenograft mouse model where miR-146a overexpressing cells decreased the tumor sizes. Downexpression of miR-146a was recently found to be caused by some epigenetic cellular events like promoter hypermethylation [20] and post-transcriptional silencing by competitive endogenous RNA (ceRNA) in NSCLC. It was also recently found that SNHG16, a competitive endogenous RNA (ceRNA), was associated with poor prognosis and upregulation in NSCLC [43]. However, the above observations were contradicted by Tan et al., who observed miR-146a overexpression both in vivo and in vitro experimental systems such as malignant lung tissue and NSCLC cell lines. He experimentally justified miR-146a as an oncomer since CHOP (DNA damage-inducible transcript 3) was targeted by miR-146a, whose downexpression has been linked to poor prognosis in lung cancer [67]. The Merlin/NF2 tumor suppressor protein level was proven to be negatively regulated by miR-146a by directly interacting with its mRNA and triggering cell proliferation, invasion, and cell migration by miR-146a in vivo cell transfection studies in A549 lung epithelial cells [68]. However, a large body of evidence proved the tumor-suppressive role of miR-146a in NSCLC. 

### 3.2. miR-146a as an Anti-Inflammatory Agent

The strong association between cancer and chronic inflammation was first discovered by Virchow in 1863 [69]. When there is an inflammatory insult such as a malignant lesion, leukocyte cells that produce a diverse variety of cytokines, chemokines, and other inflammatory molecules, will be attracted. It is a well-recognized fact that inflammation promotes cell proliferation, immune protection of malignant cells, angiogenesis, and cell metastasis [70]. There is also a strong link between COX2 and initiation of carcinogenesis [71]. One of the well-recognized miRNAs, miR-146a, is the key inflammatory signaling homeostasis and innate immunity molecules through target genes IRAK1 and TRAF6 that are downstream mediators of the IL-1α and TNFα signaling pathways. It seems that miR-146a prevents cytokine overproduction following bacterial endotoxin challenges or any inflammatory insults, and continued expression is associated with immune tolerance that fine-tunes the inflammatory system in order to stop inflammatory response overstimulation [72]. The overexpression of miR-146a in lung cells causes negative regulation of the metabolism of arachidonic acid via directly targeting the two key nodes of the inflammatory pathway: cyclooxygenase-2 (COX-2) and 5-lipoxygenase-activating protein (FLAP) [20]. This helps to prevent the overproduction of miR-146a prostaglandins E2 (PGE2) and leukotriene B4 (LTB4) that are potent inflammatory agents and promote promalignant microenvironments. Thus, it is apparent that underexpression of miR-146a leads to upregulation of COX-2 and FLAP proteins, which causes overproduction of inflammatory effector molecules such as PGE2 and LTB4 in NSCLC cell lines. This inflammatory microenvironment serves as a suitable site for the initiation of tumorigenesis. The signaling of IL-1β induces the secretions of inflammatory cytokines, such as IL-8 and RANTES. Taganov et al. found that IL-1β signaling increased miR-146a expression 24 times via the NF-κβ transcription factor [18], but when human lung alveolar epithelial cells were transfected with miR-146a mimics, significant IL-1β-induced IL-8 and RANTES inhibition was observed. While they neither targeted their release nor transcription, it was suggested that they destabilized their mechanism of translation [73]. Lambert KA et al. observed the activation of miR-146a expression as a protective negative feedback mechanism induced by inflammatory conditions to decrease inflammation. Codelivery of miR-146a and anti-inflammatory agents such as glucocorticoids enhanced their therapeutic action and thus could be proven to be a unique therapeutic strategy to reinforce the efficacy of these medications [74]. It was found by Limin that particulate matter-induced inflammation caused activation of miR-146a expression along with IL-6 and IL-8 in BEAS-2B cells. In turn, miR-146a blocked the transport of p65 towards the nucleus through inhibition of IRAK1/TRAF6 and prevented the release of IL-6 and IL-4 [75]. Thus, miR-146a also serves as a negative feedback mechanism to protect us from exogenous inflammatory agents that may trigger the development of lung cancer. Senescence is a cellular program that irreversibly prevents impaired cell proliferation and activates the release of IL-6 and IL-8, which are part of a larger senescence. Scott et al. reported the upregulation of miR-146a/146b to be allied to the senescence-associated secretory phenotype (SASP) as compared to quiescent fibroblast cells [75]. It was also observed that IL-1α signaling stimulated miR-146a/146b expression, which in turn negatively regulated IL-1α induced cytokine secretion. Therefore, it seems that miR-146a/146b expression upregulation responds to the situation of elevated inflammatory cytokine levels within a cell as a protective negative feedback, such as senescence-associated SASP activity [75]. 

### 3.3. miR-146a as a Metastatic Modulator

**Figure 3:**
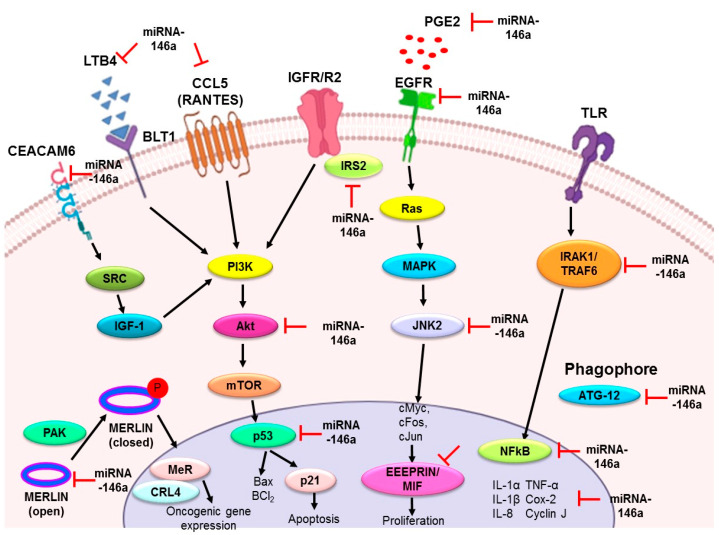
Summary of the role of miR-146a in lung cancer. The upregulation of miR-146a in lung cancer has a preventive role by acting as an antiproliferative and proapoptotic agent, anti-inflammatory agent, and metastatic modulator. Various downstream targets mentioned in the figure have been identified in different in vitro and in vivo models, justifying these properties of miR-146a. EGFR: epidermal growth factor receptor; EMMPRIN: extracellular matrix metalloproteinase inducer; MIF: macrophage migration inhibitory factor; IRS2: insulin receptor substrate 2; Merlin/NF2: moesin-ezrin-radixin-like protein/neurofibromatosis type 2; IRAK1: interleukin-1 receptor-associated kinase 1; TRAF6: tumor necrosis factor receptor (TNFR)-associated factor 6; IL-1α: interleukin 1α; IL-1β: interleukin 1β; IL-8: interleukin 8; TNF-α: tumor necrosis factor; COX-2: cyclooxygenase-2; PGE2: prostaglandin E2; LTB4: leukotriene B4; RANTES; regulated on activation, normal T cell expressed and secreted; ATG12: autophagy related 12; JNK2: c-Jun N-terminal kinases; Bcl2: B-cell lymphoma 2; CEACAM6: carcinoembryonic antigen-related cell adhesion molecule 6.

Metastasis is the migratory process of primary tumor cells to distant regions that becomes a dominant cause of death by cancer, such as in non-small cell lung cancer (NSCLC) [76,77]. Metastasis starts with a breach of physical barricades, such as the basement membrane, invading neighboring tissues and lymph nodes, and circulating into the blood and lymph. It traverses the blood vessel walls to arrive at its next destination and, finally, results in the proliferation of a secondary tumor [78,79]. A number of emerging studies found that microRNAs played a pivotal role in epithelial–mesenchymal transition (EMT), an important starting step in the development of metastasis. For instance, miR-155 enhanced the process of EMT by directly downexpressing RhoA GTPase, an important player in cell polarity, development, and maintenance of tight junctions [80]. However, after analysis of many studies, it seems that miR-146a plays a dual role (inhibitory and stimulatory) in metastasis [81]. It discourages metastasis and cell invasion by weakening NF-kB signaling and its associated targets in liver cancer [39], breast cancer [82], and colorectal cancer [83]; while its increased level in oral squamous cell carcinoma enhances stemness potential by protecting β-catenin from proteasomal degradation and downexpressing E-cadherin and CD24 transcriptions [84]. Chemoresistance and metastasis are two interwoven processes, and chemoresistance may be the trigger for metastasis [85]. Many studies found that miR-146a promoted cisplatin sensitivity or reduced cisplatin resistance and thus regressed metastasis in lung cancer via certain molecular targets already discussed in the manuscript. However, a recent study found that miR-146a significantly overexpressed, along with a reduced expression of tumor suppressor Merlin protein, and experimentally proved its direct target in lung adenocarcinoma [86] (Figure 3). 

## 4. miR-146a as a Biomarker in Lung Cancer

### 4.1. Diagnostic Potential

At the time of writing this manuscript, no study had indicated miR-146a as a single diagnostic marker in lung cancer. However, its diagnostic utility has been studied along with other miRNAs in tissue or blood from lung cancer individuals. For instance, Chen X et al. observed that miR-134, miR-146a, miR-221, miR-222, and miR-23a was greatly deregulated in lung cancer and colorectal cancer sera when analyzed through Solexa sequencing and qRT-PCR [87]. Another similar study found that miR-125a-5p, miR-145, and miR-146a in serum were significantly overexpressed in NSCLC patients with miR-146a sensitivity and a respective specificity of 92.75% and 58.57% in discriminating NSCLC patients from healthy individuals [88]. Serum miR-146a upregulation along with miR-196a-2 downregulation has been significantly associated with lung cancer patients and their polymorphisms were found to be linked to lung cancer risk in Egyptian [89] and north Indian individuals [90].

### 4.2. Prognostic Potential

As per the meta-analysis conducted by Li et al., miR-146a upregulation showed a significant association with better overall survival in most solid tumors like lung cancer, cervical cancer, ovary cancer, liver cancer, and stomach cancer, and inhibited diverse oncogenic pathways related to phospholipase C and aquaporins [52]. Additionally, downregulation of miR-146a indicated metastases with high relapse [91] and poor overall survival in lung cancer patients [19]. Contradictory to this, serum upregulation of miR-146a indicated a successful response to chemotherapy that prolonged survival in lung cancer patients [92]. Kyong-Ah Yoon et al. found that SNPs, such as rs2910164 of miR-146a and rs11614913 of miR-196a2, were positively correlated with better relapse-free survival (RFS), especially in the advanced phase of lung cancer. Furthermore, RFS showed much better improvement in individuals with higher rs2910164 and rs11614913 SNP allele frequencies [93]. These findings were contraindicated by Wang et al., who reported significantly high levels of miR-146a in the serum of NSCLC patients in comparison to the normal controls [88]. Yan-Gang Ren conducted a meta-analysis study in which lung cancer individuals found with rs11614913 in miR-196a2 and rs2910164 in miR-146a were significantly associated with lung cancer susceptibility, which appeared to contradict the earlier studies [94]. Li et al. (2017) found a similar type of observation, which was a specific elevation of miR-146a in the serum of stage I and II lung adenocarcinoma patients [52].

## 5. Potential of miRNA-146a in Lung Cancer

It has been experimentally proven both by in vivo and in vitro studies that miRNA therapy augments the chemosensitivity of malignant cells. miR-146a expression augments the therapeutic activity of EGFR TKIs like gefitinib, erlotinib, afatinib, and the monoclonal antibody cetuximab in NSCLC [95]. NSCLC cell lines experienced an improvement in cisplatin sensitivity, cell cycle arrest, cell migration suppression, and cell apoptosis by the restoration of miR-146a. Further investigation found cyclin J was directly controlled by miR-146a, which caused such effects [64]. miR-146a expression was found to be consistently lower in DDP-resistant (cisplatin) NSCLC samples and cell lines (A549, Calu-1), along with elevated levels of NF-kB activity and TNFα signaling adapter proteins like TRAF6, IRAK1. It was observed that miR-146a regulated DDP sensitivity by discouraging the activation of the NF-kB inflammatory pathway [96]. Yuwen and colleagues authenticated the same kind of work; however, they found miR-146a promoted cisplatin chemosensitivity by blocking autophagy via targeting autophagy-related protein 12 (ATG12) expression [91]. miR-146a directly targeted c-Jun N-terminal kinase (JNK2) and downregulated its mRNA level. The forced miR-146a overexpression improved the sensitivity of cisplatin in NSCLC A549/DDP cells compared to A549 cells as it promoted apoptosis but reduced the growth and invasion in A549/DDP cells via targeting p53 gene and B cell lymphoma 2 (Bcl2) gene expression [97]. Thus, miR-146a serves as a novel strategy to promote cisplatin sensitivity in NSCLC patients when used along with common chemotherapeutic agents. Carcinoembryonic antigen (CEA)-related cell adhesion molecule 6 (CEACAM6) is a glycophosphoinositol-anchored glycoprotein that belongs to the CEA family, mediating homotypic and heterotypic cell–cell interactions along with integrin receptors [98]. Zhang et al. found that the DDP-resistant (A549/DDP) cell line was significantly associated with the upregulated CEACAM6 compared to the normal lung cancer cell line (A549) [99]. When further investigation was carried out, miR-146a and miR-26a were found to promote cisplatin sensitivity and discourage the DPP-resistant malignant lung cell-associated mesenchymal phenotype by directly targeting the CEACAM6 protein post-transcriptionally [100] (Table 1).

### 5.1. miR-146a Restoration as an Adjunct Therapy to Current Therapeutic Agents

The restoration strategy exploits synthetic dsRNA structures called miRNA mimics to restore the level of diminished tumor-suppressive miRNA or small molecular drugs that release miRNA expression from inhibition within the cell. Liposomes and nanoparticles can be effectively used as the delivery vehicles for siRNA and miRNAs. This is also termed “oligonucleotide therapy” [102]. Using a combination of Let-7 and miR-34 mimics delivered to a Kras mouse model, TRP53 NSCLC showed promising results, which improved further when complemented with EGFR inhibitor (erlotinib) [103,104]. Many in vivo and in vitro studies found that miR-146a restoration by transfection with liposomal nanoparticles loaded with a synthetic mimic or incorporated into plasmids enhanced the efficacy of therapeutic drugs such as cisplatin (DPP). Since miR-146a is a potent anti-inflammatory miRNA that checks immune system overactivation, it can be used as an adjunct biologic drug with monoclonal antibody therapy and other inflammation-promoting drugs in lung cancer. miR-146a expression can also be restored by a blockade of mechanisms that diminish its expression. For example, Han et al. 2019, found SNHG16 (lncRNA) interacted with miR-146a, repressing its expression in NSCLC [43]. This condition can be resolved by making biologic drugs with a specific affinity for SNHG16 compared to endogenous miR-146a. Another study observed miRNA-146a repression by CpG promoter hypermethylation [20]. The miR-146a expression can be restored by utilization of specific DNA methyltransferase inhibitors (DNMTi). However, lack of specificity is a problem that can be resolved by promoter-specific epigenetic modifiers.

### 5.2. Potential for Small Molecule-Upregulation of miR-146a

miRNA activation is also achieved by specific chemical agents that promote or restore miRNA biogenesis or encourage miRNA-target interaction [105]. For example, Even though RNAs have long been considered as undruggable targets for small molecules, recent work indicates that miRNAs can be targeted by small molecules obtained from high-throughput screening or appropriate design [106,107,108,109]. There are many examples of small molecules that cause upregulation of specific miRNAs in cancer. Treatment with silibinin, a plant-based flavonoid, upregulated miR-494 in head and neck cancer, which downregulated ADAM10 (a disintegrin and metalloprotease domain 10) and BMI1 (B lymphoma Mo-MLV insertion region 1 homolog), resulting in inhibition of tumor growth and self-renewal properties [110]. Erismodegib, a phase III drug for medulloblastoma, upregulated the tumor-suppressor miR-128 and the expression of miR-200 family members, leading to the suppression of EMT, and suppressed the anti-apoptotic miR-21 in glioma-initiating cells [111]. An epigenetic modifier drug, decitabine (5-aza-2′-deoxycytidine), and the polyphenolic compound curcumin, both upregulated miR-145 expression [112,113]. Shen Kun-Hung found that solasodine (a natural glycoalkaloid) discouraged lung cancer metastasis by downexpressing miR-21 and MMP-2 (matrix metalloproteinase-2) [114]. Wei et al. conducted a microarray analysis, and 36 miRNAs were differentially expressed in curcumin-treated NSCLC cell lines (A549) where miR-330-5p exhibited maximum upregulation [115].

The small molecule compounds may directly interact with miR-146a or enhance its biogenesis by enhancing DROSHA and DICER activity within the cell. However, off-target effects could develop due to the global upregulation of miRNAs. Recently, many in silico and computational strategies have been found to help interaction prediction with specific small molecules. For example, Qu et al. predicted miR-146a interaction with the cytidine analog (decitabine) by utilizing an in silico prediction strategy based on the HeteSim algorithm [116]. Another study designed a new calculation model of random forest-based small molecule−miRNA association (RFSMMA) prediction based on known SM−miRNA associations in the SM2miR database, which predicted miR-146a interaction with 5-FU metabolite [117].

### 5.3. Future Perspectives and Conclusion

Despite the noninvasive nature of approaching these biomarkers in the biofluids of lung cancer subjects, many hurdles remain in the path to clinical practice. It should be noted that cell-free miRNAs (cf-miRNAs) have a nonhomogenous origin, i.e., released miRNAs originate from residing cells and endothelial cells [118,119]. This effect masks the actual number and level of miRNAs liberated by tumor-derived cells in biofluids. Also, visceral organs (lungs, liver, and kidney) that experience an elevated blood hydrostatic pressure may be a possible source of liberation [120]. There is frequent disagreement in the results obtained by research that is possibly due to different preanalytical factors like acquiring samples, storage, and small sample sizes [121]. Since a single miRNA possess a diverse array of targets governed by cellular context and targets, off-target effects are a problem in their therapeutic journey. Besides this, the exogenous miRNA unnecessarily engages with miRISC, which may distort the equilibrium of miRISC-endogenous miRNAs. Oligonucleotide therapies, such as miRNA mimics and miRNA sponges, should be cell-type specific. The synthetic double-stranded miRNAs intended to promote (miRNA mimics) or restrict (miRNA sponges) endogenous miRNA should be constituted in such a way to be freely absorbed by the cell and remain effective for the intended time period [122]. In addition to a standard and efficient delivery system for miRNAs, there is a need for mRNA target-specific activity, which can be met by deeply understanding their cellular complexity. A number of clinical trials have been carried out on autoimmune diseases [123,124] and several cancers based on miR-146a [125,126]. A clinical trial was conducted by Pavel and colleagues on former and current smokers suspected of lung cancer that were followed for a year after bronchoscopies. It was found that miRNA expression of bronchial epithelium was dysregulated in subjects with lung cancer compared to benign subjects. Among the miRNA profiles, miR-146a-5p, miR-324-5p, miR-223-3p, miR-223-5p were significantly downregulated [53]. 

Several accumulating pieces of evidence have proven that miRNA, especially miR-146a, is a crucial modulator of innate immune response sequences. Novel and exciting cancer research is involving miRNA for the detection and suppression of cancer. However, the mechanism which is adopted by these miRNAs is still unclear. MiRNAs have been used as a biomarker for cancer diagnosis due to their altered expression in various cancers compared to normal cells. The expression amount of miRNA can also be used to determine the stage of the disease, aiding in its early detection. In breast, pancreatic, and hepatocellular carcinoma, and gastric cancer, cancer cell proliferation and metastasis has been suppressed by miR-146a. This review sums up the tumor suppressor, anti-inflammatory, and antichemoresistive nature of miR-146a in lung cancer. Studies included have also suggested that miR-146a affects gene expression through different signaling pathways, viz. TNF-α, NF-κB and MEK-1/2, and JNK-1/2. Further research is required to understand the molecular mechanisms of miR-146a in lung cancer. Hence, the potential targeting of miR-146a as a diagnostic, tumor-suppressive, anti-inflammatory, and prognostic marker constitutes an important area of future research in the quest for finding a cure for lung cancer.

## Figures and Tables

**Table 1:** Experimentally validated targets of miR-146a in lung cancer.

	Molecular Target	Clinical Significance	References
miR-146a	COX2	Decreases basal prostaglandins	[35]
	Cyclin D1, Cyclin D2	Discourages cell proliferation	[63]
	IRS2	Discourages EMT/cancer progression	[65]
	IL-6, IL-8, RANTES	Prevents cytokine overproduction	[73,101]
	IRAK1, TRAF6, NF-kB	Weakens TNFα inflammatory stimuli and promotes cisplatin sensitivity	[72,101]
	Cyclin J	Promotes cisplatin chemosensitivity	[64]

COX (Cyclooxygenase); IRS2 (Insulin Receptor Substrate 2); IL (Interleukins); RANTES (Regulated upon Activation, Normal T Cell Expressed and Presumably Secreted); IRAK1 (Interleukin 1 Receptor Associated Kinase 1); TRAF6 (Tumor necrosis factor receptor associated factor 6); NF-κB (Nuclear factor kappa B).
